# Analyzing the spatial–temporal dynamics of disaster risk based on social media data: a case study of Weibo during the Typhoon Yagi period

**DOI:** 10.3389/fpubh.2025.1528199

**Published:** 2025-05-16

**Authors:** Xingqi Zou, Jiaxin Lv

**Affiliations:** Faculty of Management and Economics, Kunming University of Science and Technology, Kunming, China

**Keywords:** disaster risk diffusion, emergency knowledge dissemination, spatial–temporal dynamics, Typhoon Yagi, grounded theory

## Abstract

This paper investigates the spatial–temporal dynamics of disaster risk diffusion and the dissemination of emergency knowledge during Typhoon Yagi, one of the strongest autumn typhoons to hit China since 1949. Employing the spatial–temporal dynamics approach, the study utilizes social media data from Sina Weibo, collected through Python crawling, to analyze the diffusion process of typhoon disaster risks and the mechanism of emergency knowledge dissemination. The research examines the spatial–temporal characteristics of disaster risk diffusion and emergency knowledge dissemination, their interrelationships, and the influence of social background and geographical environment. The findings reveal that the public’s discussion on disaster risk and emergency knowledge changes over time, with distinct patterns observed during the typhoon warning, occurrence, and recovery periods. Spatially, Guangdong and Hainan provinces show the highest levels of discussion, aligning with the typhoon’s landfall locations. The study underscores the co-evolutionary nature of disaster risk diffusion and emergency knowledge dissemination, whereby the dissemination of emergency knowledge is concomitant with the diffusion of typhoon risk. This research provides a theoretical foundation for the field of disaster risk management and emergency knowledge dissemination, offering practical references for future responses to natural risks.

## 1 Introduction

Typhoons and other natural disasters pose significant challenges to human society, threatening lives, property, social economies, and ecological environments. As one of the most destructive meteorological hazards, their impacts have become a focal point for global disaster research. In September 2024, Super Typhoon Yagi, one of the strongest autumn typhoons to land in China since 1949, brought great disaster to southern China, especially the Hainan and Guangdong provinces. According to preliminary statistics, the direct economic loss of Haikou City is about 26.324 billion yuan, and the direct economic loss of Wenchang City is about 32.7 billion yuan, highlighting the severe impact of disaster risk diffusion on regional economies. In disaster risk management, analyzing the temporal and spatial dynamics of disaster risk and its influencing factors is the key to understanding the occurrence, development, and impact of disaster. This analytical framework is essential for bridging the gap between physical disaster processes and societal vulnerability. This analysis encompasses not only the physical attributes of disasters, such as the trajectory, intensity, and precipitation of typhoons, but also the implications of disasters on human society, including population distribution, economic activities, and infrastructure vulnerability. A comprehensive analysis of these factors makes it feasible to predict more precisely the potential losses that disasters might inflict, thereby providing a scientific foundation for disaster early warnings and emergency responses. As paradigmatic meteorological disasters, typhoons exhibit complex spatiotemporal risk dynamics shaped by multiple interrelated factors. Meteorological factors comprise typhoons’ intensity, path, and precipitation; geographical factors involve terrain, hydrology, and land utilization; and social factors include population density, economic activities, and the disaster resilience of communities. These multi-layered factors do not act in isolation but form an interactive system that amplifies risk propagation. These factors interact and jointly affect the risk dissemination of typhoon disasters. Hence, this paper analyzes the spatial–temporal dynamics of typhoon disaster risk dissemination and its influencing factors.

Meanwhile, the dissemination efficiency and effect of emergency knowledge directly influence the outcome of disaster response. In the era of digital connectivity, this influence has become particularly pronounced due to the proliferation of social media. During a disaster, timely and accurate information can assist people in making correct decisions, mitigating panic and chaos, and enhancing self-rescue and mutual-rescue capabilities. Hence, investigating how to effectively disseminate emergency knowledge and improve the public’s disaster awareness and response capabilities is crucial for reducing disaster losses. Against this backdrop, social platforms have emerged as significant channels for information dissemination, and their role in disaster risk management and emergency response has become increasingly prominent. Typhoons trigger secondary disasters such as strong winds, torrential rains, floods, and landslides during the landing process, causing severe casualties and property losses while provoking extensive public discussions across China. Based on this, the study takes Typhoon Yagi as a case, collects Weibo data from September 1st to 16th, 2024, through Python crawling, and discusses the diffusion process of typhoon disaster risks and the dissemination mechanism of emergency knowledge. This study examines the spatiotemporal characteristics of disaster risk diffusion and emergency knowledge dissemination, as well as their interrelationships. It also considers the influence of social background and geographical environment on these processes. The aim is to provide a theoretical foundation for research in the field of disaster risk management and emergency knowledge dissemination. In addition, the study offers feasible, practical references for future responses to natural risks.

## 2 Literature review

Natural disasters like typhoons occur unexpectedly and can significantly threaten social resources, mental well-being, public safety, and health (1). In light of the issue, Linardos et al. (2) examined how machine-learning techniques are utilized in disaster management. Zhu and Zhang (3) assessed flood risk using the random forest algorithm. In terms of investigating the spatial–temporal dynamics of disaster risk diffusion, Jiang et al. (4) studied precipitation and river flow trends in the Yangtze River Basin. Lu and Fu (5) focused on interannual variations in summer rainfall across East Asia. Additionally, Zhang et al. (6) investigated the spatial–temporal patterns of floods and droughts in China and their effects on agriculture. Liu et al. (7) looked into the spatial–temporal features of haze and particulate pollution within China. In their study, Men et al. (8) examined how natural disaster risks evolve in terms of both space and time within chemical industrial parks. Understanding the spatial–temporal evolution characteristics of disaster risks is crucial for effective disaster risk management, prompting extensive research efforts by scholars in this field. Notably, these domestic studies primarily rely on traditional meteorological and environmental data, with limited integration of social media-generated insights into public discourse during disasters.

Further, with the rapid advancement of social media, platforms like Weibo have emerged as significant channels for disseminating disaster information, offering immediate early warnings at the initial stage of a disaster, and serving as effective tools for information transfer during the post-disaster recovery period. Disaster risk diffusion pertains to the spatial and temporal spread of risks resulting from disasters, while emergency knowledge dissemination refers to the communication of information related to disaster response (9). Typhoon Yagi had rapid and severe impacts as an extreme weather phenomenon. Against this backdrop, entities such as the media and eyewitnesses promptly disseminated emergency knowledge to assist the public in getting prepared within the shortest possible time. On the Weibo platform, people extensively discussed relevant information, facilitating even faster information dissemination (10). Internationally, studies like Morelli et al. (11) and Ogie et al. (12) have systematically analyzed social media’s role in shaping risk perception and disaster recovery, but comparable domestic research remains fragmented, lacking a systematic framework to link social media data with traditional risk assessment models. Zander et al. (13) analyze how Australians use social media during natural hazards. Alexander analyzes the use of social media (blogs, messaging, sites such as Facebook, wikis, and so on) in disasters and major incidents (14). Luna and Pennock (15) point out that social media applications are dependable communication channels even when traditional methods fail. Ramakrishnan et al. (16) analyze the factors that influence the use of social media for disaster management by underserved communities. Yigitcanlar et al. (17) detect natural hazard-related disaster impact with social media analytics. Singla and Grawal (18) analyze the challenges and enablers between social media and disaster management. This study bridges this gap by providing a domestic case analysis of Weibo data, systematically comparing spatial–temporal patterns with international findings on social media’s role in disaster communication. Hence, this article analyzes the spatial–temporal characteristics of disaster risk diffusion based on social media data. In recent years, the increasing prevalence of social media platforms has transformed how information is disseminated and consumed during disasters. Researchers can gain valuable insights into public perceptions and behaviors related to disaster risks by leveraging vast amounts of user-generated content from these platforms.

## 3 Research methods

### 3.1 Research case

This study selected Typhoon Yagi, which landed in September 2024, as the research case. To capture the full lifecycle of the disaster, we collected data from Sina Weibo using Python between September 1, 2024 (when Yagi first entered public awareness), and September 16, 2024 (the recovery period after the typhoon subsided). Relevant data and content from the social media platform “Sina Weibo” were collected through Python software, such as the main text of Weibo posts, the Internet Protocol (IP) location, gender, and age of the authors. Data collection incorporated specific keyword filters to ensure relevance, including “Typhoon Yagi,” “destruction,” “disaster resistance,” “disaster relief,” and other terms related to risk diffusion and knowledge dissemination (detailed in Section 3.3.1). Based on the grounded theory (19, 20), the classification and preprocessing of Weibo texts were accomplished. Grounded theory is particularly suitable for this study as it allows for inductive theory development from unstructured social media data, aligning with our goal of exploring emergent patterns in disaster risk communication (21). Moreover, the spatiotemporal clustering method was used to generate spatiotemporal maps of disaster risk diffusion and emergency knowledge dissemination to explore the spatiotemporal distribution and its influencing factors. Typhoon Yagi, a tropical cyclone, originated in the waters east of the Philippines on the evening of September 1, 2024, and developed rapidly due to favorable atmospheric conditions, including warm sea surface temperatures and low vertical wind shear, which are critical factors for typhoon formation. By September 6 at 16:20, Typhoon Yagi made landfall along the coast of Wentian Town in Wenchang City, Hainan Province, China. At this point, it was reportedly at near-peak intensity, with sustained winds reaching significant speeds that threatened coastal communities.

During this second landfall event, it intensified into a super typhoon. The impact was severe and widespread. Numerous countries were affected by heavy rainfall and strong winds associated with Yagi’s path, including China and neighboring nations such as the Philippines and Vietnam. These adverse weather conditions led to extensive flooding and landslides in various regions. Given its severe impacts and implications for disaster management, this study selects Typhoon Yagi as a case study aimed at examining several key aspects related to natural disasters: specifically focusing on spatial–temporal characteristics regarding how disaster risks diffuse through different geographical locations over time, analyzing emergency knowledge dissemination processes among local populations, and identifying various influencing factors that contribute to both preparedness levels before such events as well as response effectiveness during recovery efforts post-disaster. This comprehensive analysis provides valuable insights that may inform future strategies for mitigating similar risks associated with extreme weather phenomena.

### 3.2 The acquisition and processing of research data

Sina Weibo is one of the highly representative social media platforms in China. It can be published through multiple terminals such as the Internet, clients, and mobile phones, enabling the posting and receiving of information at any time and place. Its audience is extremely active in China and even worldwide. In this paper, Weibo data during Typhoon Yagi is crawled using Python with specified keywords. The time range was strictly defined as September 1–16, 2024, covering the warning, occurrence, and recovery periods. To exclude irrelevant content, we filtered out posts mentioning “Yagi” in non-typhoon contexts (e.g., constellation references) and retained only posts containing predefined keywords related to disaster risk and emergency knowledge (see Table 1 for details). The main data types include the main text of Weibo posts, the longitude and latitude of the author’s IP, the posting time of Weibo, and the basic information of the author etc. Weibo posts with only topics but no substantive content and invalid data, such as references to ‘Yagi’ as a zodiac sign, were excluded. Finally, 12,478 valid contents are filtered out.

**Table 1:** Open coding.

Category	Some sentences of the original data (initial concepts)
*A*_1_: the actual situation of disaster resistance and relief	Guangdong Xuwen was also not immune to the disaster. Big trees fell, fences collapsed, and iron sheets were flying everywhere!
	From the Emergency Command Center for Typhoon Yagi in Hainan Province, we have learned that the wind circle of Typhoon Yagi has begun to affect areas such as Haikou, Chengmai, and Ding’an, and the center of the wind circle has entered Haikou, the provincial capital. Multiple residential communities in Haikou have experienced power outages and water shortages, and some residential and office buildings have had their windows shattered by the fierce winds and rainstorms. Many green trees in the city have fallen, and some coastal residential areas have experienced seawater intrusion.
	Typhoon Signal No. 8, or Severe Wind Signal, is currently in effect and is expected to last at least until noon on the 6th. Subway and light rail services will be limited from the first train, and many citizens took the subway this morning.
*A*_2_: Meteorological Description of Typhoon	The No. 8 Gale or Storm Signal, commonly called “No. 8 Typhoon Signal,” is further classified into No. 8 Northwest, Southwest, Northeast, and Southeast Gale or Storm Signals based on the blowing direction. This signal indicates that gales or storms are prevailing or expected to prevail at or near sea level in Hong Kong.
	The Shenzhen Meteorological Observatory upgraded the typhoon white warning signal for the entire city to blue at 19:00 on September 4, 2024. It is anticipated that due to the influence of Typhoon Yagi, the gusts in our city will reach above level 8.
	Meanwhile, the southeasterly wind, the easterly wind, and the northeasterly wind associated with the cold air are contributing to the formation and development of the rain belt in the coastal regions. Despite the passage of Typhoon Yagi, its influence has not yet subsided.
*A*_3_: the Perception of Disaster-related Emotions	Notwithstanding the tempestuous wind and rain, our hearts remain closely intertwined.
	Unity constitutes power! The people of Hainan, in a unified front, are working together to overcome their challenges and create a brighter future for themselves.
	The process of charging, water accumulation, and food hoarding commenced yesterday. Presently, the circumstance is serene. Such stillness is terrifying.
*A*_4_: real-time news reporting	Affected by Typhoon Yagi, the immigration management police were at the front line of disaster relief in Binqiao Township, Longzhou County, Chongzuo City, Guangxi. They were engaged in the tasks of evacuating people and rescuing materials. After working continuously for over 10 h, they were exhausted and slept on the ground. Currently, the rescue operation is still ongoing.
	Affected by the peripheral circulation of Typhoon Yagi, it is projected that from 5:20 to 07:00, the central urban area and the adjacent sea surface will experience short-term strong winds of grade 7–8, thunderstorms, and other severe convective weather, accompanied by short-term heavy precipitation, with the maximum hourly rainfall ranging from 20 to 30 millimeters.
	Typhoon Yagi is constantly drawing nearer. Today, the Central Meteorological Observatory has continuously issued the highest-level typhoon red warning.
*A*_5_: the popularization of disaster prevention knowledge	When a typhoon arrives, if one is outdoors: 1. Do not seek refuge from the wind and rain near temporary buildings, billboards, iron towers, or big trees. 2. Do not stay in low-lying areas for shelter, and select a high and solid house for refuge. 3. If driving, it is recommended to immediately drive to places such as underground parking lots for shelter.
	What are the precursors before the arrival of a typhoon? When a typhoon strikes, how should one take precautions and avoid risks indoors and outdoors? Please keep this risk avoidance guideline well.
	Safety comes first, and negligence is not allowed. Everyone should actively learn to cope with typhoons and take extra precautions.
*A*_6_: sharing of emergency materials	We express our gratitude for donating materials to address the damage and relief needs in the areas affected by Typhoon Yagi. The materials, including humanitarian rescue first aid kits and generators, have been successively loaded onto vehicles and are departing for the disaster-stricken areas. We sincerely hope that Hainan will overcome the difficulties early and restore a beautiful life.
	A host of celebrities donated supplies to the areas in Hainan stricken by Typhoon Gajab. Disasters are heartless, but humans are kind-hearted. We stand together!
	The Hainan Provincial Committee for Disaster Prevention, Mitigation, and Relief has announced the acceptance of donations for disaster relief from Typhoon Yagi. The disaster-stricken areas urgently need materials such as log grapples, generators, water pumps, chainsaws, electric saws, portable energy storage equipment, and long-lasting LED lighting fixtures. Other materials are temporarily not accepted.

### 3.3 Textual information research based on grounded theory

Grounded theory is a qualitative research approach for systematically organizing and analyzing primary data. The core of it lies in conducting inductive analysis from the data through a standardized operational process to form conceptual frameworks and theories. Grounded theory encompasses three steps: open coding, axial coding, and selective coding, and it is suitable for exploring and addressing new phenomena and issues. In the current research, a systematic theoretical framework remains inadequate within the domain of risk and knowledge co-evolution of natural disasters like typhoons. It is highly necessary to carry out in-depth research using grounded theory. This study, based on grounded theory and taking Typhoon Yagi as an example, explores its risk diffusion and knowledge dissemination in depth.

#### 3.3.1 Open coding

Open coding involves decomposing and reconfiguring the collected raw materials, assigning conceptual labels to the materials, defining concepts, and uncovering scopes through novel means. Given the extremely high degree of discussion regarding Typhoon Yagi on the Weibo platform, in this study, after setting keywords for precise information acquisition, the posting time of Weibo was controlled and screened. In the context of Typhoon Yagi’s disaster risk diffusion, 3,391 valid texts were obtained through keywords such as “destruction,” “disaster resistance,” “disaster relief,” “rainfall,” “storm,” “fear,” “scared,” “water and power outage,” “no network,” “communication disruption,” “transportation delay,” etc. In the field of emergency knowledge dissemination, 3,257 valid texts were acquired through keywords such as “disaster prevention,” “evacuation,” “stockpiling,” “reconstruction,” “government support,” “ensuring safety,” “people’s unity,” “mutual aid with love,” “temporary shelters,” etc. This study takes these texts as the raw materials for open coding. Further, to ensure the objectivity and accuracy of the coding, we specially invited two experienced professional coders to code all the original data independently. Regarding the parts with disputes or inconsistencies, the coders reached a consensus through discussions and refinements and ultimately extracted a total of 6 basic categories. The open coding categories and the sentences of some original data are presented in Table 1.

#### 3.3.2 Spindle-based encoding

Spindle coding is a further clustering analysis of the basic categories formed after open coding. It dissects their attributes and connotations, looks for correlations, and refines higher-level main categories. Based on open coding in this paper, systematic classification and analysis deeply explored the internal connections among various basic categories. Eventually, two main categories were concluded. The results of spindle coding are shown in Table 2.

**Table 2:** Spindle-based encoding.

Main category	Initial category	Category connotation
*B*_1_: disaster Risk Diffusion	*A*_1_: the actual situation of disaster resistance and relief	The actual circumstances of emergency responses, rescue operations, and post-disaster recoveries adopted by governments at all levels, relevant institutions, and individuals under the influence of Typhoon Yagi.
	*A*_2_: Meteorological Description of Typhoon	The formation and development of Typhoon Yagi and its influence on climate and environment.
	*A*_3_: the Perception of Disaster-related Emotions	Under the influence of Typhoon Yagi, the public’s emotional responses and psychological feelings toward the disaster.
*B*_2_: the dissemination of emergency knowledge	*A*_4_: real-time news reporting	Instantaneous information update on the latest developments and countermeasures regarding Typhoon Yagi.
	*A*_5_: the popularization of disaster prevention knowledge	Through various channels of knowledge dissemination, popularize the knowledge of disaster prevention and mitigation related to Typhoon Yagi among the public.
	*A*_6_: sharing of emergency materials	After the landing of Typhoon Yagi, all sectors of society coordinated resources to ensure the effective allocation and utilization of disaster relief materials.

#### 3.3.3 Selective encoding

**Figure 1:**
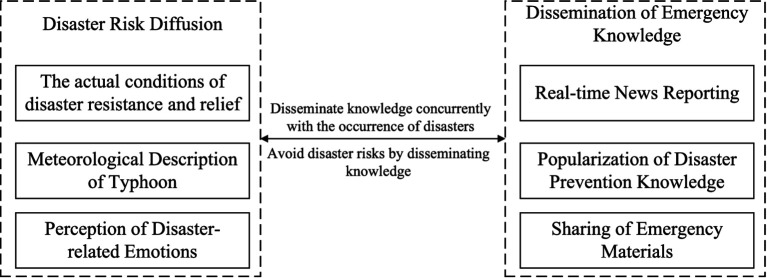
Selective encoding.

Selective coding is a further refinement of axial coding. Among the identified concept categories, find the “core category” that integrates other categories, construct a conceptual framework, integrate the research results within this framework, and utilize the collected data to verify the textual relationships. Combining materials and the CAPS theory reveals the typical relationships within the core category, clarifies the public perception behind Typhoon Yagi, and ultimately sorts out the ways of disaster risk diffusion and emergency knowledge dissemination. The typical relationship structure of the core category is shown in Figure 1.

## 4 Analyzing the spatial and temporal characteristics of disaster risk diffusion and emergency knowledge dissemination

Based on the natural development stages of Typhoon Yagi before, during, and after its landing in September 2024, this study classifies the collected Weibo data chronologically and defines them as a three-time series. (1) “Typhoon Warning Period”: from September 1st to 5th; (2) “Typhoon Occurrence Period”: from September 6th to 9th; (3) “Typhoon Recovery Period”: from September 10th to 16th. By applying the spatial–temporal dynamics method, we utilized ArcGIS software to draw the spatial distribution maps of disaster risk diffusion and emergency knowledge dissemination, aiming to investigate the degree of discussion on the topic of Typhoon Yagi among the public in different regions over time (22). This approach allows us to comprehensively analyze the spatiotemporal patterns of how information spreads and public attention shifts during different phases of the typhoon event.

### 4.1 Analysis of temporal characteristics

Based on the Grounded Theory, we relied on six initial categories (A1-A6, Table 1). We took the three periods of “Typhoon Warning Period,” “Typhoon Occurrence Period,” and “Typhoon Recovery Period” as the research timeframes. Through the proportion of each initial category in all Weibo posts, we studied the public discussion content and extent during Typhoon “Yagi.”

#### 4.1.1 The analysis of the temporal characteristics of disaster risk diffusion

**Figure 2:**
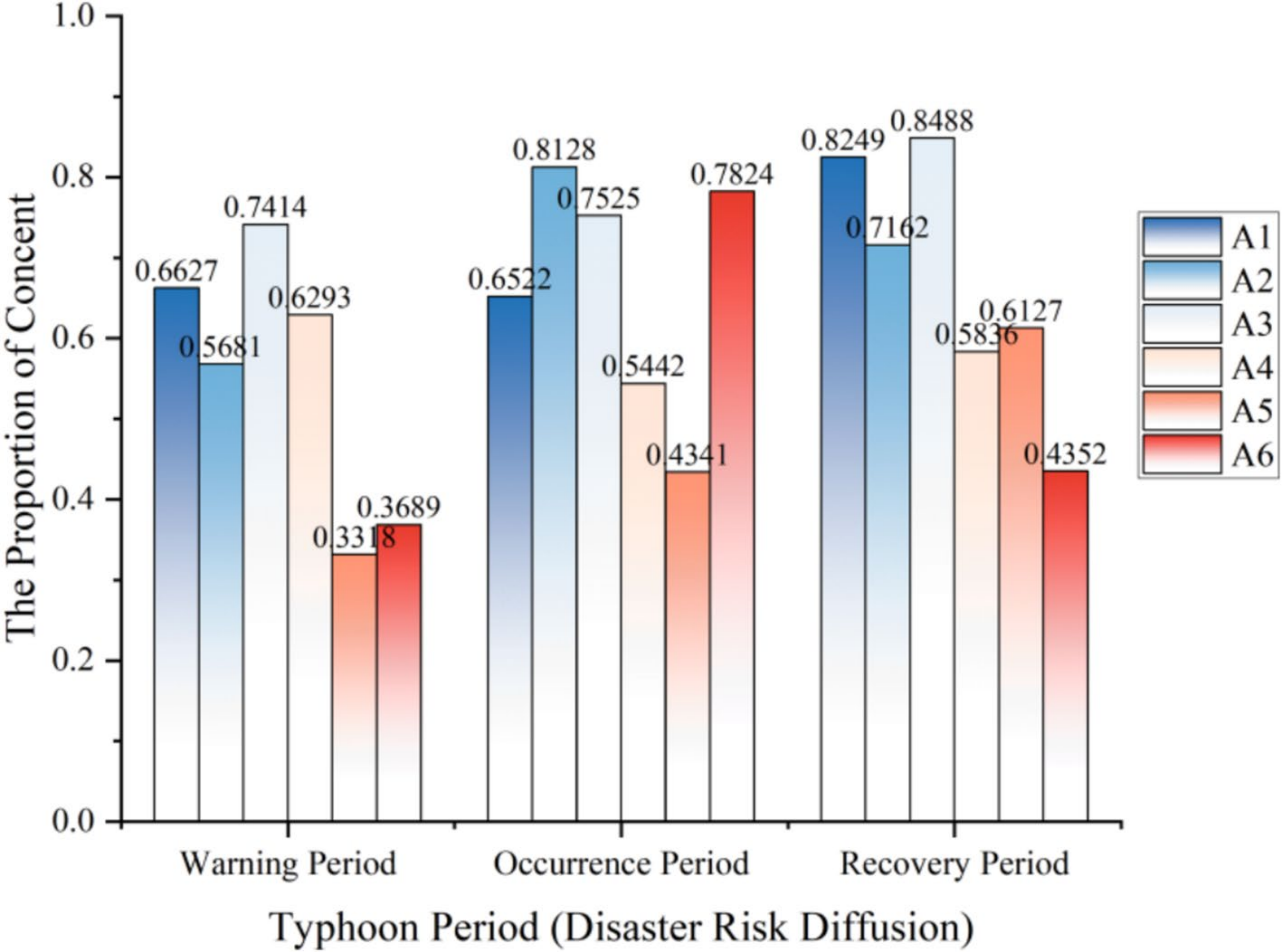
The temporal characteristics of disaster risk diffusion.

During the extreme-risk natural disaster of Typhoon Yagi, we crawled 3,391 Weibo posts related to “disaster risk diffusion.” We presented the discussion proportions of the six initial categories A1 - A6 in a tag bar chart, as shown in Figure 2.

During the occurrence of Typhoon Yagi, the discussion degrees of different themes presented diverse changing patterns over time. Among the six initial categories based on the Grounded Theory, A1 had a relatively stable discussion degree during the typhoon warning and the typhoon occurrence periods, reaching a peak of 0.8249 in the typhoon recovery period. A2 showed an overall trend of rising first and then falling, with a discussion degree as high as 0.8128 during the typhoon occurrence period. This finding aligns with gender role theory (23), where men’s greater focus on disaster relief actions (A2) reflects traditional masculine roles emphasizing problem-solving behaviors during crises. As the typhoon progressed, the public’s discussion degree regarding meteorology reached its highest. A3 was similar to A1, both stable during the warning and occurrence periods and rising during the recovery period, reflecting that the public had a stronger emotional perception of the disaster in the typhoon recovery stage.

It is worthy of in-depth study that, in the crawled “disaster risk diffusion” data, a considerable amount of content is highly correlated with “emergency knowledge dissemination.” Among the 3,391 related discussions on “disaster risk diffusion,” the discussion degree of A4 is overall relatively stable, ranging between 0.5442 and 0.6293, indicating that the public pays significant attention to news reports. A5 shows an upward trend. As the typhoon progresses, the public attaches greater importance to disseminating and learning disaster prevention knowledge. A6 has a discussion degree as high as 0.7824 during the typhoon occurrence period. Women’s stronger engagement with supply sharing (A6) corresponds to communal roles in crisis situations (24), suggesting gendered patterns in disaster response behaviors. Age-related differences were also observed, with younger users focusing more on real-time updates. At the same time, older demographics emphasized preparedness measures, consistent with protection motivation theory (25) regarding developmental differences in risk perception. While the typhoon disaster spreads, the sharing of supplies among the public flows frequently.

#### 4.1.2 The analysis of temporal characteristics of emergency knowledge dissemination

**Figure 3:**
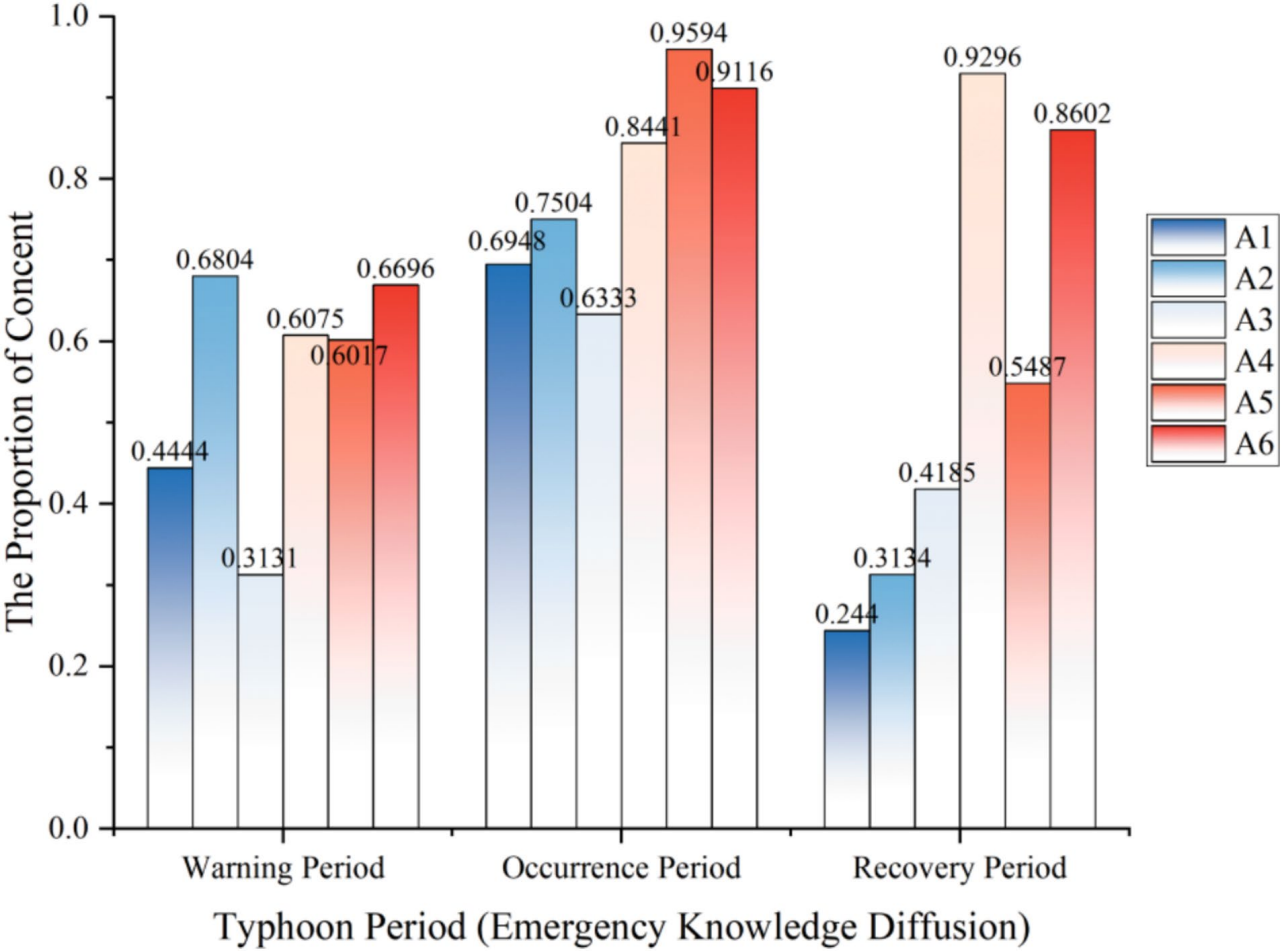
The temporal characteristics of emergency knowledge dissemination.

Similarly, we crawled 3,257 Weibo contents related to “emergency knowledge dissemination” and drew the bar chart shown in Figure 3.

Consistent with the foregoing, the content we crawled regarding “emergency knowledge dissemination” also encompasses the initial domain of “disaster risk diffusion.” The discussion intensity of A1 initially ascends and subsequently declines, attaining a peak value of 0.6948 during the typhoon occurrence period. Meanwhile, the public demonstrates high concern about real-time disaster resistance and relief situations while disseminating emergency knowledge. The distribution pattern of A2 is similar to that of A1, with the public showing the highest discussion intensity regarding meteorological changes during the typhoon occurrence period. A3 reaches its peak of 0.9296 during the typhoon recovery period. It is worth noting that it bears highly similar characteristics to the “disaster risk diffusion” data, and the public’s emotional perception during the natural disaster recovery period is the most intense. A4 exhibits an upward trend and maintains a considerable level during the typhoon recovery. Both A5 and A6 show an upward and downward trend, rising above 0.9 during the typhoon occurrence period.

### 4.2 The analysis of spatial features

The landing of Typhoon Yagi, accompanied by strong winds and torrential rains, resulted in numerous secondary disasters, such as floods and landslides, presenting considerable natural risks. Beyond the storm’s core area, the surrounding regions also endured the damage brought by the typhoon disaster. On this basis, we undertake research and discussion on the spatial characteristics of the public discourse.

#### 4.2.1 The analysis of the spatial characteristics of disaster risk diffusion

**Figure 4:**
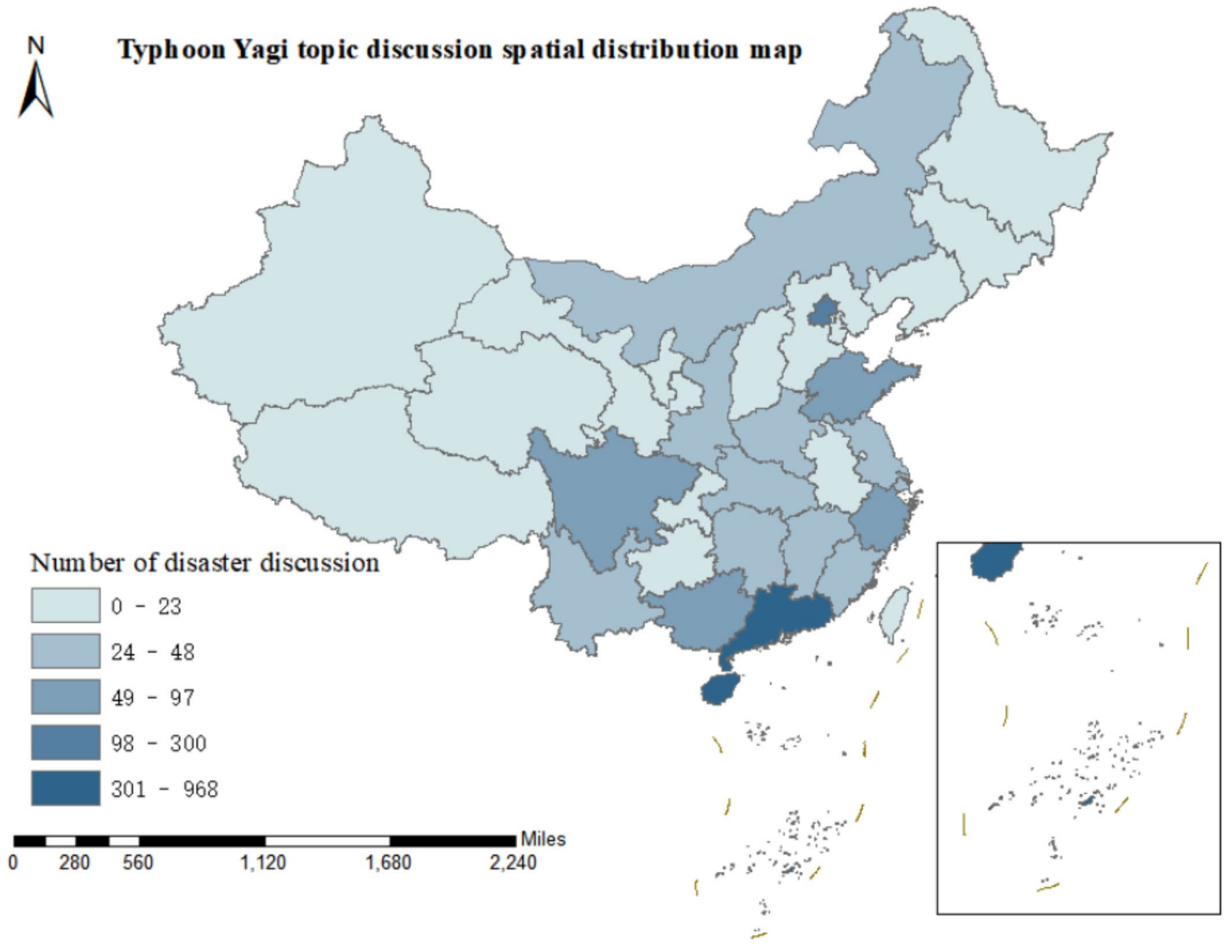
The spatial characteristics of disaster risk diffusion.

Based on 3,391 microblog posts related to “disaster risk diffusion,” we performed a visual analysis of the discussion degrees in 34 provinces, autonomous regions, municipalities directly under the Central Government, and special administrative regions of China, presenting the discussion proportions of the six initial categories A1 - A6 in the form of a map, as depicted in Figure 4.

Typhoon Yagi made landfall along the coast of Wenchang City, Hainan Province, at approximately 16:20 on September 6, 2024, and subsequently made another landfall in Xuwen County, Guangdong Province, at around 22:20 on the same day. Both landfalls were of super typhoon intensity. In the Weibo discussions related to “disaster risk diffusion,” it was observed that the general trend of the spatial distribution of the six initial categories was highly notable. Guangdong Province and Hainan Province had the highest levels of discussion, significantly surpassing those of other provincial administrative regions. This concentration reflects geographical proximity and socioeconomic factors: (1) higher population density in coastal urban areas; (2) greater economic assets at risk; (3) more developed social media infrastructure facilitating real-time information sharing. The number of discussions in Guangdong and Hainan was 891 and 968, respectively, accounting for 26.28 and 28.55% of the discussions, which combined more than half of the total. Beijing, the capital of China, ranked second with 208 discussions, indicating the concern and attention of the public in the capital toward the landfall of Typhoon Yagi. Sichuan Province, Guangxi Zhuang Autonomous Region, Shandong Province, and Zhejiang Province followed, with the number of topic discussions ranging from 49 to 97. The public in these provinces and autonomous regions paid relatively high attention to Typhoon Yagi.

#### 4.2.2 The analysis of the spatial characteristics of emergency knowledge dissemination

**Figure 5:**
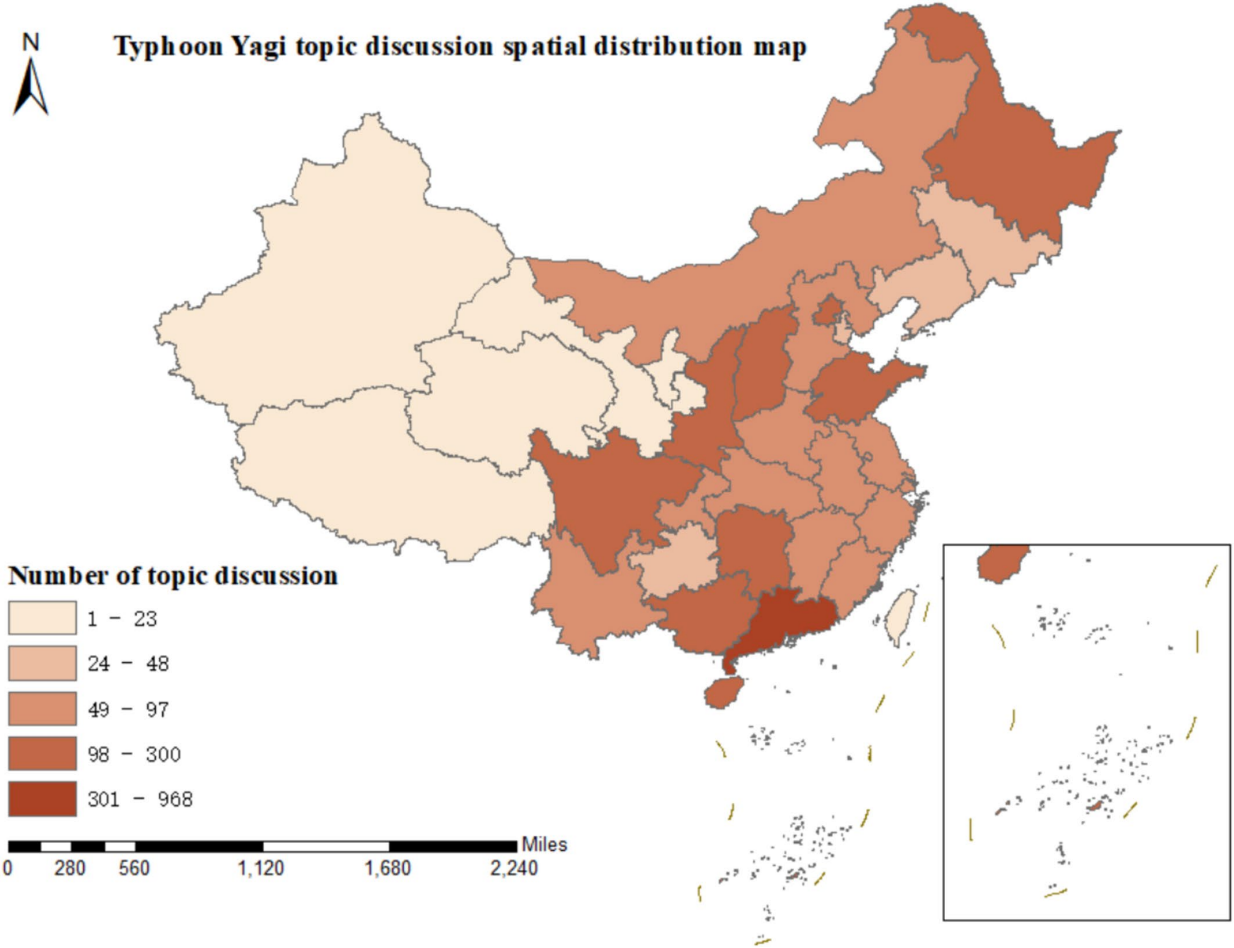
The spatial characteristics of emergency knowledge propagation.

Figure 5 presents the degree of discussion on Typhoon Yagi in 34 provinces, autonomous regions, municipalities directly under the Central Government, and special administrative regions of China based on 3,257 microblog posts related to “emergency knowledge dissemination.”

Contrary to the microblog contents related to “disaster risk diffusion,” the spatial distribution of the relevant contents of “emergency knowledge dissemination” across the provincial administrative regions of China is more evenly balanced. Guangdong Province still leads in terms of the degree of discussion, with 340 microblog posts. Hainan Province follows with 273 posts in discussion, Shanxi Province with 257, Sichuan Province with 235, Beijing with 224, and Hunan Province with 214. Most of the remaining provincial administrative regions have a relatively even number of microblog posts, ranging between 50 and 100. Thus, it can be observed that the public has highly emphasized the dissemination of emergency knowledge during Typhoon Yagi throughout various regions of China.

### 4.3 The co-evolutionary analysis of disaster risk diffusion and emergency knowledge dissemination

The previous research found a high coevolution between disaster risk diffusion and emergency knowledge dissemination during Typhoon Yagi. Most of the blog posts published by the public included relevant content about disaster risk diffusion and emergency knowledge dissemination. This co-evolution phenomenon can be understood through the social amplification of risk framework (26), where risk information and protective knowledge mutually reinforce through social networks. For example, “Blue V” news blogger “Sanxiang City Daily” published a micro-blog related to Typhoon Yagi at 21:27 on September 9, 2024, which covers the disaster situation and weather description of Typhoon Yagi landing in Hainan Province, real-time news reports and common-sense interpretation of disaster avoidance, and the spread of relevant news about disaster relief and emergency supplies. Furthermore, he expressed gratitude to the relief workers for their timely assistance. There are countless Weibo like this. The practice shows that the spread of the typhoon Yagi disaster risk is accompanied by the spread of emergency knowledge. Before Typhoon Yagi landed, local governments and meteorological departments in China released information on the spread of disasters, such as typhoon track, wind speed, and rainfall through various channels, as well as guidance and suggestions on disaster prevention and risk aversion, to help the general public prepare in advance and reduce potential risks and losses brought by Typhoon Yagi. Effective emergency knowledge dissemination can help the public quickly access disaster-related information and grasp response measures.

## 5 Analysing influencing factors of disaster risk diffusion and emergency knowledge diffusion

Through the above analysis, we found that social background, geographical location, and other factors impact the dissemination of disaster risk and emergency knowledge about Typhoon Yagi. To conduct follow-up research on the influencing factors, we randomly selected 5,830 microblogs with the keyword “Typhoon Yagi” from September 1 to 16, 2024.

### 5.1 Social background factor

**Figure 6:**
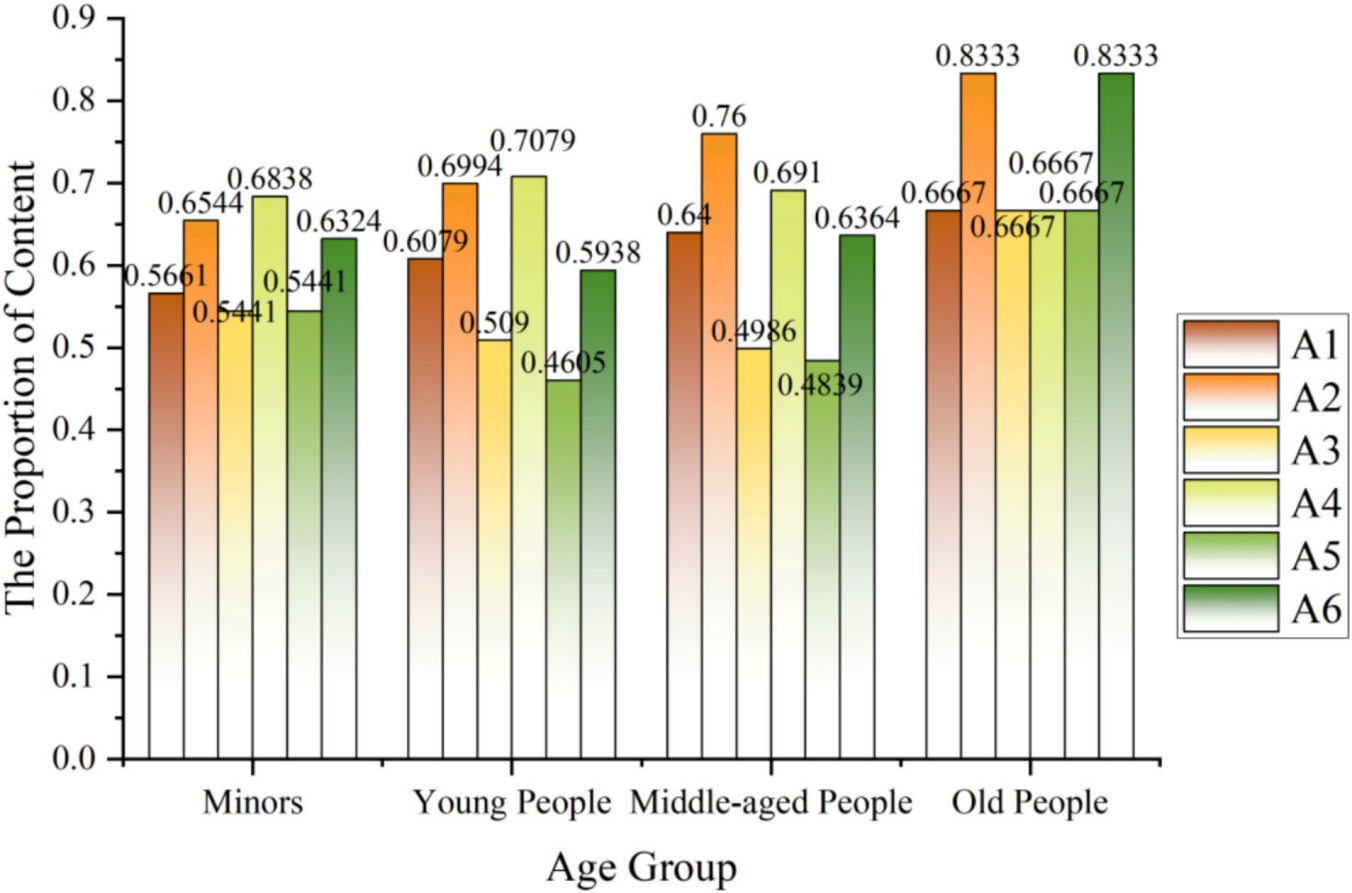
The analysis of age.

Based on the gender and birthday information of Weibo users, we studied the factors affecting the social background of disaster risk diffusion and emergency knowledge dissemination. Weibo can only crawl out the user’s birthday, and the birthday information of some users is kept secret. Therefore, we converted the user’s birthday into the user’s age, excluded the information of users born before 1924, and finally got 1937 valid data. We refer to the age division of the United Nations World Health Organization and identify minors (0–17 years), young people (18–65 years), middle-aged (66–79 years), and older adult (80–99 years). The research found that minors, young people, and middle-aged people are most concerned about real-time news reports, typhoon meteorological descriptions, and emergency materials sharing, respectively. At the same time, the older adult are more concerned about typhoon meteorological descriptions and emergency materials sharing, reflecting that people of different ages have different degrees of emphasis on other aspects, as shown in Figure 6.

**Figure 7:**
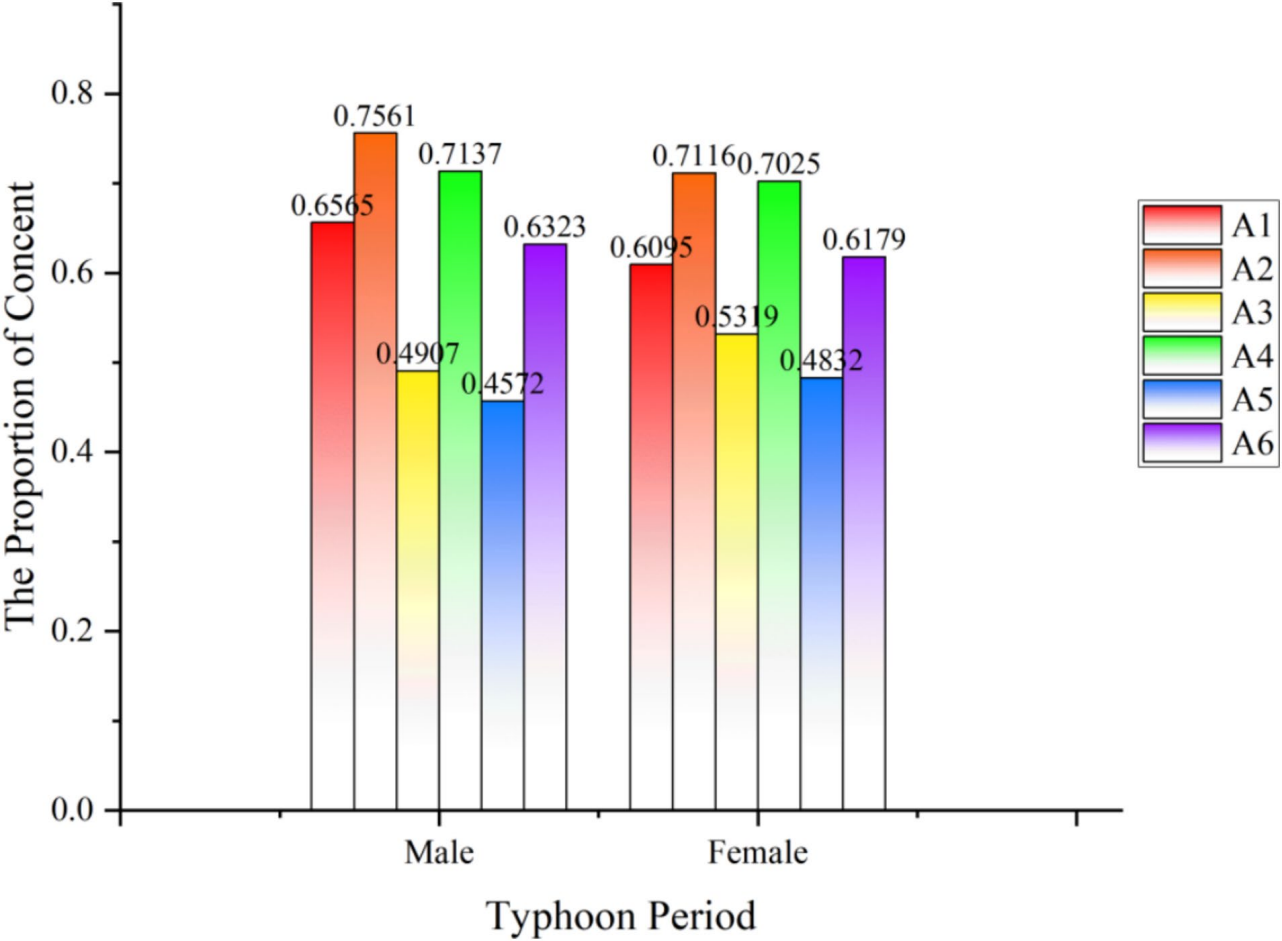
The analysis of sex.

Regarding gender, 2,358 valid records for males and 3,429 valid for females, as shown in Figure 7. During Typhoon Yagi, both men and women received high attention, and both strongly discussed typhoon meteorological descriptions and real-time news reports. In addition, men pay more attention to disaster relief, while women pay more attention to emergency material sharing, which shows that men and women pay more attention to risk events in a relatively consistent state, which is also different due to gender differences.

### 5.2 Geographical environment factor

**Figure 8:**
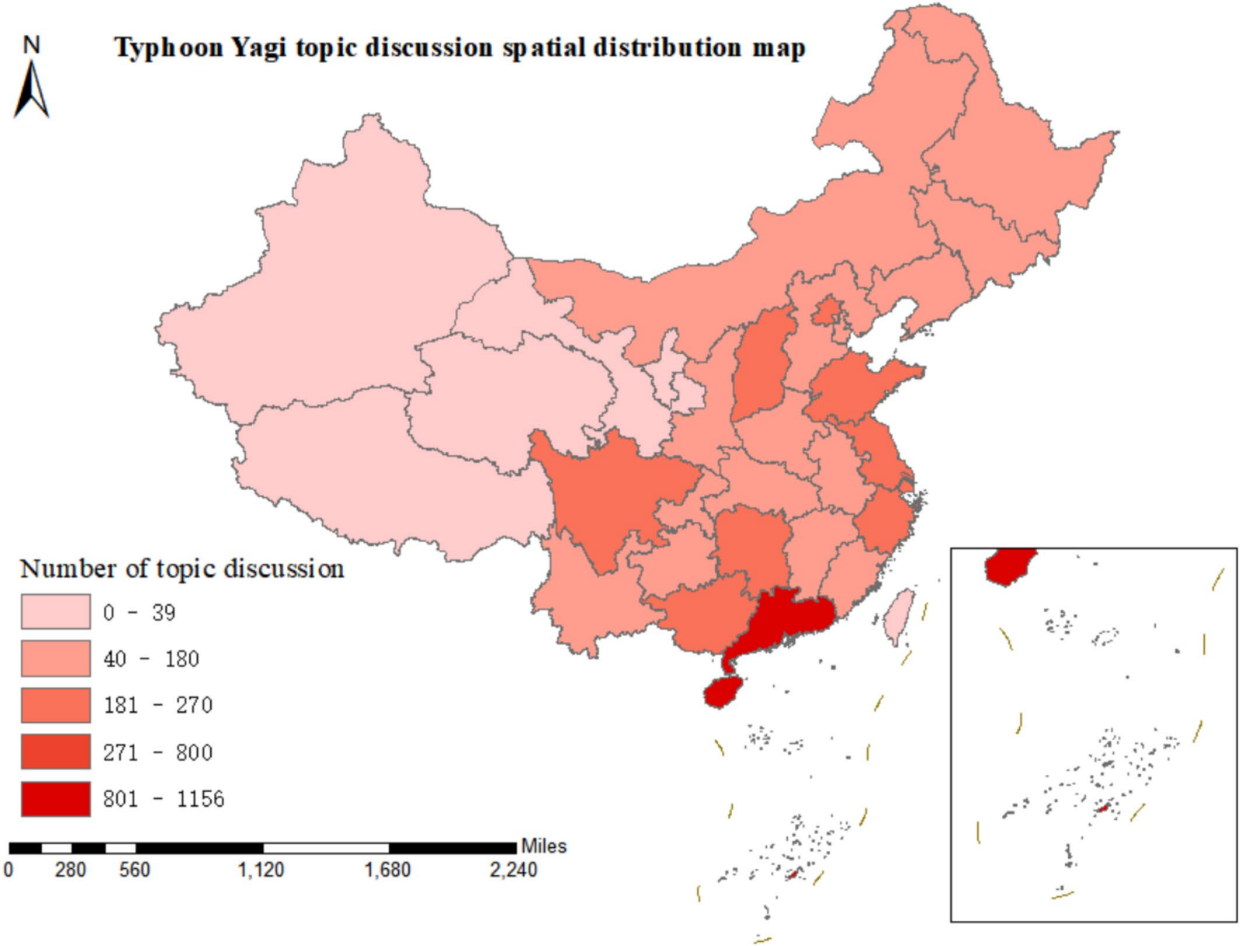
The geographical environment factor.

The previous research found that residents of Guangdong and Hainan provinces had the highest discussion about Typhoon Yagi. Based on this, we used ArcGIS software to draw a map of China for 5,830 IP territories with the keywords Typhoon Yagi related microblog from September 1 to 16, 2024, as the control group of this study, as shown in Figure 8. The results show that Guangdong Province and Hainan Province still have the highest number of discussions in China. After entering the South China Sea, Typhoon Yagi strengthened rapidly, upgraded from a typhoon to a strong typhoon in just 15 h, and finally developed into a super typhoon and gradually landed in Hainan Province, Guangdong Province, and other places. The degree of public concern for the typhoons in this region is consistent with the natural phenomenon, indicating that geographical and environmental factors have a greater impact on spreading disaster risk and emergency knowledge.

## 6 Conclusion

Under the natural disaster perspective of Typhoon Yagi, we use the microblogging platform to screen text data related to disaster risk diffusion and emergency knowledge dissemination, and explore their spatial and temporal distribution characteristics and influencing factors through data analysis. The spatial–temporal dynamics analysis shows that the public’s discourse on Yagi tends to evolve over time. Additionally, the focal point of microblog content shifts by the progression of the typhoon warning period, typhoon occurrence period, and typhoon recovery period. Regarding space, Guangzhou and Hainan Provinces showed the highest level of discussion, which aligns with the natural phenomenon of Typhoon Yagi’s landfall. As highlighted in Cutter et al.’s vulnerability framework, this spatial concentration is further explained by socioeconomic factors, such as population density and social media infrastructure. The public discussion of Typhoon Yagi on Weibo shows strong co-evolutionary characteristics. While experiencing or witnessing Typhoon Yagi as a natural disaster, the public received explanations of emergency knowledge from official media, typhoon witnesses, and other channels. It minimized the risks arising from the landfall of Typhoon Yagi by learning about emergencies, sharing information, and exchanging materials.

While this study provides valuable insights into disaster communication patterns, several methodological limitations should be acknowledged. First, our data processing pipeline, though systematically implemented through Python-based text mining and geospatial analysis, could benefit from more detailed documentation of the technical parameters and filtering criteria applied during Weibo data cleaning and classification. Second, the map generation process, while effectively visualizing spatial patterns, may not fully capture localized anomalies. These limitations highlight opportunities for future research to adopt advanced natural language processing algorithms and high-resolution spatial models and expand data collection to platforms like WeChat and Douyin to enhance generalizability.

This paper is of great significance in studying the co-evolution of risk and knowledge and provides theoretical support for knowledge dissemination under natural disasters. This study’s practical implications include guiding emergency management agencies to prioritize social media monitoring during disasters and design gender/age-sensitive communication strategies. Although Typhoon Yagi has passed, the damage caused to the affected areas is long-lasting, and the government and the public should be more concerned about helping the affected areas in the future. At the same time, our data source has a certain degree of limitation; in the follow-up study, we can use WeChat public number, Jittery, Xiaohongshu, and other mainstream media software to broaden the platform of data crawling to provide more channels of data support for the conclusion.

## Data Availability

The raw data supporting the conclusions of this article will be made available by the authors, without undue reservation.
